# Common marmoset (*Callithrix jacchus*) personality, subjective well-being, hair cortisol level and *AVPR1a*, *OPRM1*, and *DAT* genotypes

**DOI:** 10.1038/s41598-018-28112-7

**Published:** 2018-07-06

**Authors:** Miho Inoue-Murayama, Chihiro Yokoyama, Yumi Yamanashi, Alexander Weiss

**Affiliations:** 1 Wildlife Research Center of Kyoto University, Kyoto, Japan; 2 Wildlife Genome Collaborative Research Group, National Institute for Environmental Studies, Tsukuba, Japan; 3 Laboratory for Brain Connectomics Imaging, RIKEN Center for Biosystems Dynamics Research, Kobe, Japan; 4 Center for Research and Education of Wildlife, Kyoto City Zoo, Kyoto, Japan; 5 School of Philosophy, Psychology and Language Sciences, Department of Psychology, The University of Edinburgh, Edinburgh, United Kingdom; 6 Scottish Primate Research Group, Edinburgh, United Kingdom

## Abstract

We studied personality, subjective well-being, and hair cortisol level, in common marmosets *Callithrix jacchus*, a small, cooperatively breeding New World monkey, by examining their associations with one another and genotypes. Subjects were 68 males and 9 females that lived in the RIKEN Center for Life Science Technologies. Personality and subjective well-being were assessed by keeper ratings on two questionnaires, hair samples were obtained to assay cortisol level and buccal swabs were used to assess *AVPR1a*, *OPRM1* and *DAT* genotypes. Three personality domains—Dominance, Sociability, and Neuroticism—were identified. Consistent with findings in other species, Sociability and Neuroticism were related to higher and lower subjective well-being, respectively. Sociability was also associated with higher hair cortisol levels. The personality domains and hair cortisol levels were heritable and associated with genotypes: the short form of *AVPR1a* was associated with lower Neuroticism and the AA genotype of the A111T SNP of *OPRM1* was related to lower Dominance, lower Neuroticism, and higher hair cortisol level. Some genetic associations were not in directions that one would expect given findings in other species. These findings provide insights into the proximate and ultimate bases of personality in common marmosets, other primates and humans.

## Introduction

Studies of common marmosets *Callithrix jacchus*, a small New World monkey species, have identified similarities between the domains along which their personality traits are organized and the ‘personality domains’ of more cognitively advanced species. Specifically, like humans^1^ and chimpanzees^2^, common marmosets were found to possess a personality domain (Conscientiousness) that captures individual differences in self-control, predictability, and tameness/social appropriateness^3,4^. Studies of common marmoset personality also found that the reliabilities of ratings were within the range of those identified in humans^3,4^, evidence for sex and age differences^3^, evidence that their personality is influenced by the social environment^5^, and evidence for associations between personality domains and both behaviours^4,5^ and the serotonergic system in the brain^6^.

We built on these previous findings by determining the degree to which the correlates of common marmoset personality resembled those of other nonhuman primates and humans. To do so, we tested for associations between common marmoset personality and subjective well-being. In humans^7^ and nonhuman primates^8–12^, subjective well-being is associated with lower levels of traits related to anxiety, vigilance, and fear, and higher levels of traits related to sociability and activity.

We also tested for associations between common marmoset personality and hair cortisol level, which is a biological marker of chronic stress^13^. Across nonhuman primates and other animals, glucocorticosteroid levels have been associated with a ‘reactive coping style’^14^. For example, a study found that in rhesus macaques, blood cortisol levels at different parts of the day varied with personality: macaques higher in excitability had lower afternoon cortisol; macaques lower in confidence had lower morning cortisol and cortisol levels did not decline in the afternoon; macaque sociability was not related to cortisol^15^. Another study found that brown capuchin monkeys with higher baseline levels of blood cortisol engaged in play behaviours with conspecifics^16^. Studies of hair cortisol and temperament have yielded similar results: higher levels were associated with higher reactivity^17^ and less social engagement and less play in rhesus macaques^18^, and lower novelty-seeking in vervet monkeys^19^.

Finally, we sought to determine whether the genetic causes of personality and hair cortisol level in marmosets were like those identified in other nonhuman primates and humans. We therefore tested whether individual differences in personality or hair cortisol level, like those of humans^20,21^ and those of nonhuman primates^22–29^, were heritable and whether they were associated with any of four genetic polymorphisms described below.

The first polymorphism that we examined was the arginine vasopressin receptor 1 A (*AVPR1a*) gene. In humans, the RS1 and RS3 microsatellite regions in the upstream of *AVPR1a* were found to be in linkage disequilibrium with a concern for appropriateness and with sibling conflict^30^. Moreover, longer forms of *AVPR1a* have been associated with greater pair-bonding and higher levels of socially appropriate behaviour in humans and other primates^28,30–38^. The second and third were the A111T and T329C polymorphisms of the μ-opioid receptor gene (*OPRM1*). In marmosets, the A variant of *OPRM1* A111T was associated with reduced cyclic adenosine monophosphate (cAMP) inhibition by endogenous opioids and two opioid agonists: morphine and D-Ala^2^, *N*-MePhe^4^, Gly-ol (DAMGO)^39^. In rhesus macaques, the G variant of a similar polymorphism (*OPRM1* C77G) was functionally similar to *OPRM1* A111T: it was associated with lower cAMP inhibition in response to endogenous opioids, and the agonists morphine, DAMGO, and fentanyl^39^. The G variant in rhesus monkeys also coded for opioid receptors that had a greater affinity for endogenous opioids, but not the opioid agonists naloxone or buprenorphine^40^. Behaviourally and physiologically, this variant in rhesus macaques was associated with greater maternal attachment^41,42^ and both higher aggression and lower basal cortisol^40^. In humans, the A variant of *OPRM1* A118G was associated with lower cAMP inhibition in response to morphine, but not to endogenous opioids or other opioid agonists^39^. This polymorphism was thus functionally similar to the *OPRM1* polymorphisms in marmosets and rhesus macaques, but differed in that its inhibitory action was specific to morphine. A previous study found no evidence for an association between attachment and the G variant in humans; however, this study found evidence a significant interaction: subjects that carried the G variant exhibited high levels of fearful attachment, but among subjects that reported high levels of maternal care, the G variant was associated with lower levels of fearful attachment^43^. The second *OPRM1* polymorphism, T329C, was novel, identified in common marmosets, and we report on it in this paper. The fourth polymorphism was the 3′ untranslated region of the dopamine transporter gene (*DAT*)^44^. Associations between *DAT* genotype and behaviour were recently reported in robust capuchin monkeys and in common marmosets^45^. Marmosets that carried the 10-base pair repeat allele were more flexible in their responses to an operant foraging task^45^. Humans that carry the 9-base pair repeat allele of *DAT1* were more angry and impulsive^46^.

## Results

### Interrater reliabilities of items

The interrater reliabilities of the 54 personality questionnaire items are presented in Table S1. The items ‘reckless’, ‘inventive’, ‘unemotional’, ‘persistent’, ‘quitting’, ‘anxious’, and ‘unperceptive’ had interrater reliabilities below zero and so were not used in further analyses. The interrater reliabilities of individual ratings for the remaining items ranged from 0.03 for ‘depressed’ to 0.46 for both ‘solitary’ and ‘protective’ (mean = 0.20, *SD* = 0.11). The interrater reliabilities of mean ratings for these items ranged from 0.08 for ‘depressed’ to 0.69 for both ‘solitary’ and ‘protective’ (mean = 0.38, *SD* = 0.16).

The interrater reliabilities of individual ratings were 0.14, 0.07, 0.14, and 0.10, respectively, for the subjective well-being items relating to the balance of positive and negative moods, pleasure derived from social interactions, the ability to achieve goals, and global satisfaction. The interrater reliabilities of mean ratings for these items were 0.30, 0.16, 0.30, and 0.23, respectively.

### Exploratory factor analyses

#### Personality

A parallel analysis and examination of the scree plot suggested that there were three factors that accounted for 56% of the variance. An oblique (promax) rotation revealed two correlations between factors, *r*_I,II_ = −0.22 and *r*_I,III_ = 0.23, that were modest in size, and one medium-sized correlation, *r*_II,III_ = −0.40. Comparison of the promax- and varimax-rotated structures indicated that they did not differ much: all three congruence coefficients exceeded 0.94. Given these results, we interpreted the varimax-rotated structure (Table 1). The promax-rotated structure is presented in Table S2.

**Table 1:** Exploratory factor analysis with varimax rotation.

	Factor			*h* ^2^
	Dominance	Sociability	Neuroticism	
Defiant	**0.86**	−0.04	−0.11	0.748
Stingy/greedy	**0.85**	−0.13	−0.03	0.744
Jealous	**0.84**	−0.10	−0.05	0.725
Aggressive	**0.82**	−0.24	−0.12	0.752
Dominant	**0.81**	−0.21	−0.06	0.701
Irritable	**0.79**	−0.04	0.19	0.668
Bullying	**0.79**	−0.17	−0.08	0.652
Excitable	**0.76**	0.03	0.38	0.719
Impulsive	**0.73**	−0.02	**0.46**	0.756
Submissive	−**0.70**	0.09	0.30	0.591
Friendly	−**0.69**	**0.56**	−0.17	0.820
Gentle	−**0.69**	**0.54**	−0.15	0.786
Cool	−**0.69**	0.11	−0.37	0.620
Disorganized	**0.68**	−0.02	0.39	0.616
Erratic	**0.67**	−0.16	0.38	0.626
Active	**0.57**	**0.50**	−0.05	0.577
Manipulative	**0.56**	0.07	−**0.47**	0.539
Conventional	−**0.49**	**0.45**	−0.18	0.477
Predictable	−**0.48**	0.06	−0.21	0.276
Distractible	**0.42**	−0.08	0.34	0.297
Thoughtless	**0.40**	0.00	0.25	0.226
Helpful	−0.36	**0.76**	−0.27	0.774
Solitary	0.26	−**0.74**	0.35	0.742
Imitative	−0.10	**0.72**	−0.12	0.544
Dependent/follower	−0.31	**0.72**	0.08	0.617
Protective	−0.34	**0.71**	−0.20	0.664
Individualistic	0.39	−**0.71**	0.26	0.723
Independent	**0.41**	−**0.70**	0.05	0.668
Sociable	−**0.52**	**0.68**	−0.37	0.875
Sympathetic	−**0.46**	**0.65**	−0.14	0.661
Affectionate	−**0.53**	**0.62**	−0.03	0.662
Playful	0.22	**0.56**	−0.01	0.361
Sensitive	−**0.47**	**0.55**	−0.29	0.610
Curious	0.38	**0.53**	−0.06	0.428
Inquisitive	0.33	**0.48**	−0.05	0.346
Lazy	−0.34	−**0.47**	0.35	0.460
Innovative	0.14	0.38	−0.04	0.166
Timid	0.04	−0.15	**0.74**	0.580
Stable	−0.39	0.37	−**0.62**	0.674
Autistic	−0.04	−0.13	**0.62**	0.402
Fearful	0.18	−0.01	**0.56**	0.351
Vulnerable	−0.36	−0.17	**0.52**	0.424
Intelligent	−0.22	**0.44**	−**0.49**	0.487
Clumsy	0.02	−0.24	**0.45**	0.261
Depressed	−0.15	−**0.41**	**0.44**	0.386
Decisive	−0.26	0.34	−0.34	0.300
Cautious	0.06	0.00	0.28	0.082
Proportion of variance	0.27	0.18	0.11	

Note. *h*^2^ = communality.

The factors that emerged resembled those found in a behavioural assay of 12 common marmosets from the same facility^6^. The items that loaded on the first factor indicated that subjects that were high on this factor were aggressive, assertive, and lacked forethought and focus. This factor resembled domains labelled Dominance, Assertiveness, or Confidence found in common marmosets^3,4^ and in other primates^2,9,12,47–49^, and so we named it Dominance. The items loading on the second factor indicated that subjects that were high on this factor were prosocial, gregarious, active, and explored their environment. This factor therefore resembled, in parts, a domain labelled Agreeableness in one study of common marmosets^3^ and domains labelled Extraversion and Agreeableness in another study of marmosets^4^. Similar domains, i.e., those characterised by high Agreeableness and Extraversion, have been identified in rhesus macaques, other macaque species, western lowland gorillas and brown capuchin monkeys^9,47,49,50^. The domains identified in these studies were labelled ‘Friendliness’ or ‘Sociability’. We therefore named this factor Sociability. The items loading on the third factor indicated that subjects that were high on this factor were emotionally unstable, emotionally and socially withdrawn, fearful and easily upset by other marmosets. Previous analyses of common marmoset personality ratings did not yield a similar personality domain^3,4^. However, behavioural assays in two studies, including a study of 12 common marmosets at the same facility, revealed a similar domain^4,6^. To be consistent with previous studies, for example, of orangutans^12^, chimpanzees^2^ and brown capuchin monkeys^47^, we named this factor Neuroticism.

#### Subjective well-being

A parallel analysis and scree plot indicated that the four subjective well-being items defined a single factor. This factor accounted for 64% of the variance. All four items had salient loadings on this factor: >0.99, 0.83, 0.81, and 0.46 for global life satisfaction, the balance of positive versus negative moods, the ability to achieve goals, and the amount of pleasure derived from social interactions, respectively.

### Interrater and internal consistency reliabilities of factors

For Dominance, Sociability, and Neuroticism, respectively, the interrater reliabilities of individual ratings were 0.41, 0.48, and 0.28, the interrater reliabilities of the mean ratings were 0.64, 0.70, and 0.50, and the standardized Cronbach’s alphas were 0.95, 0.93, and 0.83. The interrater reliabilities of individual ratings, of mean ratings, and the standardized Cronbach’s alpha, for subjective well-being were 0.15, 0.32, and 0.84, respectively.

### Correlations between personality domains and subjective well-being

Subjective well-being was significantly associated with higher Sociability, lower Neuroticism, but not Dominance (Table 2). The significant associations survived adjusting for multiple tests. At the item level, Sociability was significantly associated with higher ratings on all four subjective well-being items and all four associations survived correction for multiple tests; Neuroticism was significantly associated with lower scores on all four items, but the association between Neuroticism and the item concerning happiness derived from social interactions did not survive correction for multiple tests (Table 2). Dominance was not significantly associated with any of the subjective well-being items.

**Table 2:** Pearson correlations between personality domains and subjective well-being items and subjective well−being.

	Dominance			Sociability			Neuroticism		
	*L95*	*r*	*U95*	*L95*	*r*	*U95*	*L95*	*r*	*U95*
Balance of moods	−0.35	−0.14	0.09	**0.24**	**0.44**	**0.60**	−**0.52**	**−0.34**	−**0.12**
Pleasure from social interactions	−0.32	−0.10	0.12	**0.42**	**0.59**	**0.72**	−0.49	−0.30	−0.08
Goals	−0.27	−0.05	0.18	**0.25**	**0.45**	**0.61**	−**0.56**	**−0.38**	−**0.17**
Global satisfaction	−0.33	−0.12	0.11	**0.29**	**0.49**	**0.64**	−**0.61**	**−0.45**	−**0.25**
Subjective Well−being	−0.34	−0.12	0.11	**0.43**	**0.59**	**0.72**	−**0.61**	**−0.44**	−**0.24**

Note. Values in boldface significant after Bonferroni correction for multiple tests. *L95* = lower 95% confidence interval, *U95* = upper 95% confidence interval.

We carried out robustness checks (details in the Supplementary Results) to test whether any of these associations were adversely affected by the male skew in this sample or by the large number of subjects that were not reared by their parents or not housed with other marmosets. These checks revealed a significant interaction, which suggested that the association between Neuroticism and the balance of positive and negative moods was stronger among normally reared and housed subjects. However, this interaction did not survive correction for multiple tests. The other associations between personality and either the subjective well-being factor or items did not differ as a function of backgrounds.

### Personality domains, subjective well-being and hair cortisol level

Hair cortisol level was significantly associated with Dominance (*r* = −0.30, 95% *CI* = −0.53 to −0.03, *P* = 0.030) and Sociability (*r* = 0.43, 95% *CI* = 0.19 to 0.63, *P* = 0.001), but only its association with Sociability survived adjusting for multiple tests. Neuroticism was associated with lower levels of cortisol, but this association was not significant (*r* = −0.27, 95% *CI* = −0.50 to 0.00, *P* = 0.054). The association between hair cortisol level and subjective well-being was significant (*r* = 0.33, 95% *CI* = 0.07 to 0.55, *P* = 0.016), but did not survive adjustment for multiple tests.

We carried out robustness checks like those used to test the robustness of associations between personality domains and subjective well-being. Details of these robustness checks can be found in the Supplementary Results. The associations did not differ as a function of the subjects’ backgrounds.

### Genotyping

For *AVPR1a*, 73 out of 77 subjects were successfully genotyped and 10 alleles (202, 204, 206, 208, 210, 212, 214, 216, 218, and 222 base pairs) were identified; heterozygosity was 0.852. We characterized alleles with 202 to 210 base pairs (frequency: 0.582) as “short” and alleles with 212 to 222 base pairs (frequency: 0.418) as “long”: 13 subjects were homozygous for the long allele (LL), 25 were homozygous for the short allele (SS) and 35 were heterozygous (SL).

For *OPRM1*, 75 out of 77 samples were successfully genotyped. Heterozygosity was 0.500 for A111T: 21 subjects were homozygous for A, 19 were homozygous for T and 35 were heterozygous. Heterozygosity was 0.440 for T329C: 9 marmosets were homozygous for T, 35 were homozygous for C and 31 were heterozygous.

For *DAT*, 76 out of 77 samples were successfully genotyped. We identified 3 alleles with 420 (frequency: 0.355), 460 (0.526), 500 base pairs (0.118) corresponding to alleles including 9, 10 and 11 repeats in the previous report^51^. Heterozygosity was 0.583. We characterized 420 base pair alleles as “short” and the 460 and 500 base pair alleles as “long”: 30 subjects were homozygous for the long allele, 8 were homozygous for the short allele and 38 were heterozygous.

We used the HWAlltests function from the HardyWeinberg package in R^52,53^ to test whether the genotypes were in Hardy-Weinberg equilibrium. The *P*-values for all tests were above 0.05, and so the alleles were in Hardy-Weinberg equilibrium (Table S3).

### Animal models

We used the deviance information criterion to identify which of three models for each test of association had the best balance of model fit and model parsimony. The model fit index and heritability estimate associated with each model is presented in Table S4. Trace plots for the models with the best balance of model fit and parsimony (the lowest deviance information criterion) did not signal problems with autocorrelation and density plots indicated that the distribution of estimates about the mean was approximately normal (Supplementary Datafile).

#### Personality

For *AVPR1a*, among males with complete personality data, 12 had the LL genotype, 32 had the SL genotype and 20 had the SS genotype. For *OPRM1* A111T, among males with complete date, 19 had the AA genotype and 47 had the TT (n = 18) or the AT genotype (n = 29). For *OPRM1* T329C, among males with complete data, 31 had the CC genotype and 35 had the TC (n = 27) or the TT genotype (n = 8). For *DAT*, among the 67 males with complete data, 25 had the LL genotype and 42 had the SL (*n* = 34) or SS (*n* = 8) genotype.

The best model representing the Dominance and *AVPR1a* association indicated that parent-reared males that lived with conspecifics, i.e., males with a normal background, were significantly higher in Dominance (Table 3). This model included two significant interactions: SL and SS males with a normal background were lower in Dominance than LL males; SL and SS males with a non-normal background were higher in Dominance than LL males. The best model representing the Dominance and *OPRM1* A111T association indicated that AA males were significantly lower in Dominance than TT and AT males (Table 3). The parameters of the best model representing the association between Dominance and *OPRM1* T329C and the best model representing the association between Dominance and *DAT* did not include any significant parameters (Table 3). The median heritability of Dominance across the four models was 0.40.

**Table 3:** Animal models for Dominance.

	estimate	l−95% CI	u−95% CI	N_effective_	P
	*AVPR1a*				
**Model 1**					
Intercept	50.23	43.26	57.60	10000	<0.001
SL vs. LL	0.14	−7.25	7.23	10517	0.98
SS vs. LL	−4.33	−12.09	4.18	10718	0.29
**Model 2**					
Intercept	50.01	42.92	56.87	10000	<0.001
Normal	−1.39	−4.08	1.46	10000	0.32
SL vs. LL	1.01	−6.19	8.42	10000	0.79
SS vs. LL	−4.02	−12.48	3.92	9897	0.33
Model 3*					
Intercept	51.48	44.74	58.47	10000	<0.001
Normal	**6.44**	**1.13**	**11.60**	**9586**	**0.014**
SL vs. LL	0.56	−6.38	8.29	10361	0.88
SS vs. LL	−5.94	−13.81	1.80	10000	0.13
Normal x SL vs. LL	−**11.43**	−**18.12**	−**4.85**	**10000**	**0.001**
Normal x SS vs. LL	−**9.37**	−**16.53**	−**2.63**	**10000**	**0.008**
	***OPRM1*** **A111T**				
Model 1*					
Intercept	51.04	47.02	55.07	10000	<0.001
AA	−**5.60**	−**10.87**	−**0.26**	**10000**	**0.041**
**Model 2**					
Intercept	51.16	47.20	55.32	9547	<0.001
Normal	−0.61	−3.16	1.95	10392	0.65
AA	−5.45	−10.95	−0.07	10000	0.048
**Model 3**					
Intercept	51.07	46.97	55.18	9374	<0.001
Normal	−0.49	−3.49	2.54	9700	0.74
AA	−5.23	−11.22	1.02	9558	0.086
Normal x AA	−0.40	−6.86	5.46	10000	0.90
	***OPRM1*** **T329C**				
**Model 1**					
Intercept	50.22	45.77	54.71	10000	<0.001
CC	−2.76	−8.79	3.34	10000	0.35
**Model 2**					
Intercept	50.37	45.63	54.75	9944	<0.001
Normal	−0.82	−3.39	1.77	10000	0.54
CC	−2.59	−8.62	3.39	9625	0.39
Model 3*					
Intercept	50.72	46.05	55.34	10000	<0.001
Normal	−2.95	−6.40	0.52	9880	0.097
CC	−4.08	−10.15	2.46	10000	0.20
Normal x CC	4.73	−0.38	9.85	10578	0.069
	***DAT*** **420**				
**Model 1**					
Intercept	47.47	42.54	52.35	10000	<0.001
420 bp carrier	2.23	−2.62	7.41	10000	0.37
Model 2*					
Intercept	47.62	42.66	52.55	10000	<0.001
Normal	−1.49	−4.17	1.16	10000	0.27
420 bp carrier	2.64	−2.22	7.83	9524	0.31
**Model 3**					
Intercept	48.00	43.00	53.20	10000	<0.001
Normal	−2.65	−6.74	1.48	10015	0.20
420 bp carrier	2.10	−3.07	7.52	9503	0.43
Normal x420 bp carrier	2.03	−3.49	7.56	10000	0.45

Note. The personality variable was scaled so that it had a mean of 50 and standard deviation of 10. N_effective_ = effective sample size. * = best model.

The best model representing the Sociability and *AVPR1a* association indicated that there were two interactions: carriers of the short allele that had a normal background were higher in Sociability than LL males; Sociability did not vary as a function of genotype among males that did not have a normal background (Table 4). The best model representing the association of Sociability and *OPRM1* T329C indicated that normally reared males were higher in Sociability (Table 4). The best models representing association between Sociability and *OPRM1* A111T and the association between Sociability and *DAT* did not include any significant parameters (Table 4). The median heritability of Sociability across these four models was 0.62.

**Table 4:** Animal models for Sociability.

	estimate	l−95% CI	u−95% CI	N	P
	*AVPR1a*				
**Model 1**					
Intercept	45.45	38.63	52.74	10000	<0.001
SL vs. LL	5.26	−2.14	12.13	10000	0.14
SS vs. LL	7.16	−0.85	15.32	10000	0.086
**Model 2**					
Intercept	45.85	38.87	52.84	10909	<0.001
Normal	2.52	0.06	5.14	10000	0.053
SL vs. LL	3.67	−3.90	10.64	10000	0.31
SS vs. LL	6.58	−1.01	14.83	9931	0.10
Model 3*					
Intercept	44.64	37.91	51.36	10000	<0.001
Normal	−3.10	−8.11	1.76	10000	0.21
SL vs. LL	4.98	−1.75	12.31	10000	0.16
SS vs. LL	7.53	0.10	15.43	10000	0.056
Normal x SL vs. LL	**6.47**	**0.23**	**12.80**	**10000**	**0.046**
Normal x SS vs. LL	**8.89**	**2.42**	**15.52**	**10395**	**0.009**
	***OPRM1*** **A111T**				
**Model 1**					
Intercept	49.37	45.09	53.84	10000	<0.001
AA	3.28	−2.30	8.50	10000	0.22
Model 2*					
Intercept	49.09	44.87	53.52	10000	<0.001
Normal	2.36	−0.06	4.79	10523	0.057
AA	2.58	−2.96	7.75	10000	0.34
**Model 3**					
Intercept	48.57	44.47	52.98	10000	<0.001
Normal	3.57	0.96	6.45	10000	0.013
AA	4.83	−0.80	10.81	10000	0.10
Normal x AA	−5.13	−10.78	0.53	10326	0.077
	***OPRM1*** **T329C**				
**Model 1**					
Intercept	49.97	45.62	55.02	10000	<0.001
CC	1.76	−4.46	8.15	10000	0.57
**Model 2**					
Intercept	49.52	44.61	53.98	10000	<0.001
Normal	2.52	0.18	4.91	10000	0.037
CC	1.46	−4.71	7.59	9719	0.65
Model 3*					
Intercept	49.32	44.83	53.95	10000	<0.001
Normal	**3.40**	**0.13**	**6.64**	**10000**	**0.039**
CC	2.20	−4.03	8.75	10000	0.49
Normal x CC	−2.01	−6.55	3.01	10321	0.40
	***DAT*** **420**				
**Model 1**					
Intercept	50.06	45.30	55.01	10000	<0.001
420 bp carrier	1.23	−3.50	5.94	10000	0.60
**Model 2**					
Intercept	49.85	45.18	54.73	10000	<0.001
Normal	2.09	−0.39	4.58	10000	0.10
420 bp carrier	0.65	−4.20	5.21	10000	0.79
Model 3*					
Intercept	50.19	45.11	55.04	10000	<0.001
Normal	1.13	−2.57	5.08	9543	0.56
420 bp carrier	0.25	−4.60	5.05	10000	0.91
Normal x420 bp carrier	1.71	−3.43	6.85	9562	0.51

Note. The personality variable was scaled so that it had a mean of 50 and standard deviation of 10. N_effective_ = effective sample size. * = best model.

The best model representing the Neuroticism and *AVPR1a* association indicated that males that carried the short allele were lower in Neuroticism (Table 5). The best model representing the Neuroticism and *OPRM1* A111T association indicated that males that had a normal background and that possessed the AA genotype were significantly lower in Neuroticism (Table 5). This model also included a significant interaction: the association between the AA genotype and lower Neuroticism was larger among males that did not have a normal background (Table 5). The best models representing associations between Neuroticism and *OPRM1* T329C and between Neuroticism and *DAT* indicated that Neuroticism was lower among males with a normal background (Table 5). The median heritability of Neuroticism across these four models was 0.62.

**Table 5:** Animal models for Neuroticism.

	estimate	l−95% CI	u−95% CI	N	P
	*AVPR1a*				
**Model 1**					
Intercept	58.48	52.20	64.87	10000	<0.001
SL vs. LL	−12.35	−18.98	−5.73	10000	<0.001
SS vs. LL	−9.14	−16.17	−1.69	10000	0.014
**Model 2**					
Intercept	57.30	51.23	63.82	10000	<0.001
Normal	−3.47	−5.81	−1.09	10000	0.005
SL vs. LL	−9.61	−15.84	−2.91	9667	0.005
SS vs. LL	−7.89	−14.93	−0.72	10373	0.031
Model 3*					
Intercept	57.95	51.60	64.25	10000	<0.001
Normal	−0.33	−4.98	4.30	9624	0.89
SL vs. LL	−**10.07**	−**16.46**	−**3.40**	**10000**	**0.003**
SS vs. LL	−**8.59**	−**15.76**	−**1.44**	**10000**	**0.019**
Normal x SL vs. LL	−4.21	−10.24	1.65	10000	0.16
Normal x SS vs. LL	−4.45	−10.66	1.86	9501	0.15
	***OPRM1*** **A111T**				
**Model 1**					
Intercept	51.23	47.10	55.36	9523	<0.001
AA	−6.01	−11.47	−0.86	10044	0.028
**Model 2**					
Intercept	51.59	47.44	55.52	10191	<0.001
Normal	−3.72	−6.04	−1.42	10000	0.002
AA	−4.87	−9.94	−0.01	10000	0.051
Model 3*					
Intercept	52.19	48.12	55.97	10000	<0.001
Normal	−**5.10**	−**7.69**	−**2.58**	**10000**	<**0.001**
AA	−**7.41**	−**13.03**	−**2.06**	**10000**	**0.006**
Normal x AA	**5.80**	**0.36**	**11.14**	**10000**	**0.034**
	***OPRM1*** **T329C**				
**Model 1**					
Intercept	47.68	43.09	52.25	10000	<0.001
CC	2.85	−3.13	8.99	10000	0.36
Model 2*					
Intercept	48.25	43.97	52.69	10000	<0.001
Normal	−**4.17**	−**6.60**	−**1.99**	**10000**	<**0.001**
CC	3.50	−2.18	9.27	10000	0.23
**Model 3**					
Intercept	48.25	43.88	52.86	10000	<0.001
Normal	−4.12	−7.37	−1.07	10000	0.01
CC	3.53	−2.51	9.60	10000	0.25
Normal x CC	−0.11	−4.76	4.38	9620	0.96
	***DAT*** **420**				
**Model 1**					
Intercept	49.70	44.86	54.44	10117	<0.001
420 bp carrier	−1.36	−6.14	3.39	10000	0.56
Model 2*					
Intercept	49.97	45.28	54.63	10000	<0.001
Normal	−**3.23**	−**5.75**	−**0.90**	**10000**	**0.009**
420 bp carrier	−0.57	−5.19	3.94	10000	0.81
**Model 3**					
Intercept	50.26	45.53	55.10	10000	<0.001
Normal	−4.19	−8.04	−0.53	10000	0.028
420 bp carrier	−0.93	−5.55	4.00	10000	0.69
Normal x420 bp carrier	1.70	−3.60	6.45	10000	0.51

Note. The personality variable was scaled so that it had a mean of 50 and standard deviation of 10. N_effective_ = effective sample size. * = best model.

#### Hair cortisol level

The best model representing the association between hair cortisol level and *AVPR1a* did not include any significant parameters (Table 6). The best model representing the association between hair cortisol level and *OPRM1* A111T indicated that males with the AA genotype had significantly higher levels (Table 6). This model also included a significant interaction: the association between hair cortisol level and genotype was weaker among males that had a normal background (Table 6). The best models representing associations between hair cortisol level and *OPRM1* T329C and *DAT* did not include significant parameters (Table 6). The median heritability of hair cortisol level across the four models was 0.36.

**Table 6:** Animal models for cortisol.

	estimate	l−95% CI	u−95% CI	N	P
	*AVPR1a*				
Model 1*					
Intercept	49.81	42.25	57.03	9740	<0.001
SL vs. LL	−1.06	−8.75	6.87	10000	0.80
SS vs. LL	5.32	−3.32	13.92	10000	0.22
**Model 2**					
Intercept	49.88	42.67	57.71	10350	<0.001
Normal	−0.15	−3.43	3.10	10000	0.93
SL vs. LL	−1.03	−8.95	7.39	10387	0.80
SS vs. LL	5.31	−2.92	14.42	10000	0.22
**Model 3**					
Intercept	49.52	42.12	57.36	9654	<0.001
Normal	−1.14	−7.22	5.04	10000	0.71
SL vs. LL	−2.25	−10.69	6.57	9691	0.62
SS vs. LL	5.87	−2.43	15.30	9619	0.20
Normal x SL vs. LL	3.37	−4.68	12.12	10000	0.42
Normal x SS vs. LL	−0.32	−8.73	8.02	10000	0.93
	***OPRM1*** **A111T**				
**Model 1**					
Intercept	49.20	44.83	53.91	10000	<0.001
AA	5.84	−0.92	12.84	10000	0.10
**Model 2**					
Intercept	49.41	44.99	54.27	10306	<0.001
Normal	−1.17	−4.29	2.04	9686	0.47
AA	6.37	−1.02	13.18	10000	0.084
Model 3*					
Intercept	48.64	44.55	52.82	9288	<0.001
Normal	0.49	−2.84	3.64	10332	0.78
AA	**13.10**	**4.74**	**21.26**	**9663**	**0.004**
Normal x AA	−**10.53**	−**18.68**	−**2.14**	**9788**	**0.012**
	***OPRM1*** **T329C**				
Model 1*					
Intercept	52.43	47.74	56.96	10000	<0.001
CC	−3.94	−11.14	3.19	10000	0.27
**Model 2**					
Intercept	52.56	47.66	57.40	10000	<0.001
Normal	−0.33	−3.53	2.94	10000	0.84
CC	−3.88	−10.77	3.78	9679	0.28
**Model 3**					
Intercept	52.76	48.00	57.36	10000	<0.001
Normal	−1.46	−5.38	2.50	10000	0.46
CC	−5.65	−13.51	2.62	10000	0.16
Normal x CC	3.48	−3.26	10.14	10000	0.30
	***DAT*** **420**				
**Model 1**					
Intercept	49.20	43.73	54.76	9198	<0.001
420 bp carrier	3.21	−2.74	9.01	10000	0.28
Model 2*					
Intercept	49.47	44.14	55.33	9307	<0.001
Normal	−1.56	−4.82	1.78	10000	0.35
420 bp carrier	3.92	−1.96	9.90	10374	0.19
**Model 3**					
Intercept	49.16	43.91	54.93	10000	<0.001
Normal	0.53	−4.16	5.53	10000	0.83
420 bp carrier	4.90	−1.43	10.94	9715	0.12
Normal x420 bp carrier	−3.85	−10.01	2.78	10000	0.23

Note. The personality variable was scaled so that it had a mean of 50 and standard deviation of 10. N_effective_ = effective sample size. * = best model.

## Discussion

The mean of the item interrater reliabilities was close to that found in previous studies of common marmosets (0.26^4^ and 0.20^54^) and squirrel monkeys (0.24^55^) and it was within the range of (but lower than) the mean interrater reliabilities of items from human personality questionnaires (0.31^56^). Factor analysis revealed a Dominance, Sociability, and Neuroticism domain. These domains had moderate to good interrater reliabilities and excellent internal consistency reliabilities. Sociability and Neuroticism were associated with higher and lower subjective well-being, respectively, and Sociability was associated with higher hair cortisol levels. These associations did not differ as a function of background.

Dominance, Neuroticism, and Sociability resembled domains that emerged from behavioural observations of common marmosets at the RIKEN Center for Life Science Technologies^6^. Similar personality domains were also identified in a study of common marmoset personality that used behavioural tests: Dominance, Neuroticism (reversed), or a combination of high Dominance and low Neuroticism resembled the Boldness domain from the earlier study and aspects of Sociability resembled the Exploration domain from the earlier study^5^. Moreover, studies of personality in common marmosets that used a similar questionnaires found Dominance domains under the names ‘Extraversion’^4^ and ‘Assertiveness’^3^ and a domain named ‘Agreeableness’^3,4^ the latter being similar to the Sociability domain that we report. Unlike two recent rating-based studies of common marmosets^3,4^, we did not find a Conscientiousness factor. One possible explanation for this difference between our study and the previous studies is that the rearing or housing conditions in our study may not have allowed for the behavioural expression of Conscientiousness. Although studies of some primate species, such as chimpanzees^57^, have found that the type of housing or living conditions have limited effects on the personality domains that emerge, social influences do appear to influence marmoset personality^5^. Another possibility is that the interactions between raters and subjects in our study were such that traits related to Conscientiousness were less salient to the raters.

The associations between marmoset personality domains related to sociability and emotional stability and higher subjective well-being are consistent with findings in chimpanzees^10,11,58^, orangutans^12^, rhesus macaques^9^, brown capuchin monkeys^8^, and humans^7^. These findings suggest that the association between personality and subjective well-being is phylogenetically old.

The associations between hair cortisol level and personality in marmosets not consistent with previous findings. For one, there was no association between Neuroticism and cortisol level, which was surprising given the association between hair cortisol levels and a reactive temperament in rhesus macaques^17^. Moreover, we found a positive association between Sociability and hair cortisol levels, which was not what one would expect based on studies of rhesus macaques^15,18^, vervet monkeys^19^, and brown capuchin monkeys^16^ (the evidence for such an association in humans is mixed^59,60^). One possible reason why our findings contradict those from studies of other nonhuman primates is that they reflect differences in the socioecology of common marmosets and other species. The finding that the association between cortisol and rank varies across species with different social structures^61^ supports this possibility. Further studies of the association between personality and hair cortisol levels in other callitrichids, which are also cooperative breeders, and New World monkey species that are not, are needed to test this possibility.

Dominance, Sociability, Neuroticism, and hair cortisol levels were heritable. Our analyses also revealed that there were associations between *AVPR1a* and *OPRM1* A111T polymorphisms and our personality and cortisol phenotypes: the short form of *AVPR1a* was associated with lower Neuroticism and the AA genotype of *OPRM1* A111T was related to lower Dominance, lower Neuroticism, and higher hair cortisol levels. These analyses also revealed that about 40 to 60% of the variation in personality and cortisol levels was heritable.

The long version of the *AVPR1a* gene has been associated with pair-bonding, socially appropriate behaviours, and Conscientiousness^28,30–38,62^. Our finding that the short genotype was associated with lower Neuroticism, which included traits relating to repetitive stereotyped behaviours (‘autistic’) and those related to fearfulness in social situations (‘timid’), is not consistent with the prior findings described above. One possible explanation is that our findings reflect the background (rearing and housing) of many of the subjects. However, because there was no significant genotype by background interaction for Neuroticism, this is unlikely. Another possibility is that because the *AVPR1a* polymorphism in common marmosets is at the intron whereas it is at the promoter region in other species^28,30–38^, and thus this polymorphism has different effects on behaviour.

Compared to carriers of the T allele of *OPRM1* A111T, marmosets homozygous for the A allele were lower in Dominance, lower in Neuroticism, and had higher hair cortisol levels. The findings concerning Dominance and cortisol in our study are opposite to those from a study of rhesus macaques, which found that a functionally similar polymorphism was related to lower cortisol and higher aggression^40^. On the other hand, our finding of an association between this polymorphism and lower Neuroticism was consistent with the association between a functionally similar polymorphism and lower cortisol in rhesus macaques^40^. These differences may be attributable to or reflect the fact that, unlike rhesus macaques, where dominant and subordinate individuals have similar cortisol levels, among common marmosets, subordinate individuals have lower cortisol levels^61^. Another possibility is that the environment experienced by the subjects in our study influenced the relationship between cortisol and personality. This explanation is consistent with our finding of a significant interaction effect such that the association between *OPRM1* A111T and cortisol is stronger among animals that were hand-reared and/or solitary housed. Another possibility is that these differences reflect the use of hair cortisol measures, which captures cortisol levels over long periods of time, in our study and the use of basal plasma cortisol in the study of rhesus macaques^40^. To rule out one or the other of these possibilities requires studies that compare associations between functionally similar *OPRM1* polymorphisms, personality, and hair cortisol level across species that differ in whether or how rank is related to stress.

There were limitations to our study. For one, our sample size was relatively small. Personality traits are influenced by many genes with very small effects^63^, and so there is a risk that the genetic associations identified in this study were false positives. The second and third limitations were that the subjects were predominantly (88.3%) male and just under a third (31.2%) of the subjects were hand-reared or not housed with other marmosets. A fourth limitation was that detailed information on social dominance was available only for four subjects, meaning that we could not test whether these associations varied across individuals that differed in social rank. We addressed the second and third limitation by conducting robustness checks. For the phenotypic associations, these checks involved excluding females and testing whether associations differed between subjects with and without a normal background. For the genetic associations, we fit models for the males only, tested whether genotype × rearing interaction terms were significant and only interpreted genetic associations when where there was a significant main effect of genotype. These checks suggested that the phenotypic associations were not unduly influenced by differences in background. However, they indicated that housing and/or rearing differences may have obscured the association between the *AVPR1a* genotype and both Dominance and Sociability. To address these limitations would require a large study, ideally on traits that have genome-wide significance in humans or related species, of a mixed-sex sample with subjects that were normally reared and that lived in naturalistic groups.

Although there is a need to be cautious in interpreting the genetic associations that we found, our findings highlight how suited marmosets are for studying personality and affect. For one, this study and others^3–5^ have demonstrated that common marmoset personalities can be described by domains resembling those found in other primates, including humans. In addition, as in humans and non-cooperatively breeding primates^7–12,20,22–28^, the domains are heritable, and there are consistent associations between personality domains and subjective well-being. On the other hand, the relationships between personality and both cortisol and genotypes were often different from what one would expect. It is thus possible that these domains arose independently in common marmosets and so are analogues instead of homologues. Future studies that compare how common marmoset personality is associated with fitness, social and non-social behaviours, and genetic and neurophysiological variables to such associations in other species will allow us to better understand how functionally similar personality traits evolve in species that evolved in different social and ecological environments.

## Methods

### Subjects

Subjects were 77 common marmosets (68 males and 9 females). At the time they were rated, subjects ranged in age from 1.5 to 15.1 years (mean = 6.0, SD = 2.6). Subjects were housed in the RIKEN Center for Lifestyle Technologies in Kobe, Japan, and comprised 61 born at RIKEN, 6 supplied by CLEA Japan Inc. (Tokyo, Japan), and 10 supplied by Japan Wild Animal Laboratory Limited (Amami, Japan). Subjects sourced from other facilities had lived in the RIKEN Center for Lifestyle Technologies for at least 3 years prior to this study.

There was variation in subjects’ rearing histories and whether subjects were single housed or socially housed. At the time their personalities were rated, 52 parent-reared and 12 non-parent-reared subjects were housed in a family group (n = 4), with an opposite-sex marmoset for breeding (n = 10) or with male peers (n = 50). The remaining subjects–4 parent-reared, 8 hand-reared, 1 with an unknown rearing history–were single-housed. Further details about animal husbandry, housing, and diet are presented in the Supplementary Methods.

This study complied with the current laws of Japan, including the Act on Welfare and Management of Animals. Experimental and husbandry procedures were performed in accordance with the Guidelines for Conducting Animal Experiments of RIKEN, where the study was conducted, and in accordance with the recommendations of the ARRIVE (Animal Research: Reporting of *In Vivo* Experiments) guidelines^64^. All procedures were approved by the Animal Care and Use Committee of the Kobe Institute of RIKEN (MAH21-10-8).

### Ratings

Personality ratings were made on the 54-item Hominoid Personality Questionnaire^11,65^. Subjective well-being ratings were made on a four item questionnaire that has been described in previous studies^10^. For both questionnaires, raters were instructed to respond to each question using a 7-point scale and to not discuss their ratings. Further details about the questionnaires including a link to the questionnaires are provided in the Supplementary Methods.

We collected 199 ratings. Keepers (two men and one woman) completed Japanese-language rating forms for subjects that they knew well. Thirty-two subjects were rated by two keepers and 45 were rated by all three keepers. The keepers had known the subjects they rated for between 1.08 and 9.83 years (mean = 4.34 years). There were no missing data.

### Cortisol assays

Samples were collected from subjects by cutting tail hairs with scissors. Hair was cut at the skin surface and collected. To the extent possible, hair was cut from the same location in every subject. The length of 240 hair samples taken from 24 subjects (10 samples each) ranged from 19.9 to 27.5 mm (mean = 23.05 mm). To assay cortisol concentrations in hair we used an approach adapted from studies of chimpanzees (Supplementary Methods)^66,67^. Marmoset hair is much thinner than chimpanzee hair and thus contains about 100 times more cortisol^68–70^. Intraassay variability was 4.41% on average and interassay variabilities were 4.99% for high control and 8.05% for low control.

### Genotyping

A buccal swab from each subject was kept in a 90% ethanol solution until DNA extraction. DNA was extracted by DNeasy Blood and Tissue kit (Qiagen, CA, USA). For *AVPR1a*, we surveyed the (GT)n microsatellite in the first intron locating the 1009^th^ nucleotide from the transcription start site, and the 39^th^ nucleotide from the first intron start site. PCR amplification was conducted in a 10 μl (the total volume) reaction mixture containing a DNA template, each primer, LA *Taq*, dNTPs, and GC buffer I (TaKaRa, Shiga, Japan). After denaturing DNA samples at 94 °C for 1 min, we set up 35 cycles of 94 °C for 30 seconds, 60 °C for 30 seconds, 74 °C for 1 minute, and a final extension at 74 °C for 10 minutes. The length of the PCR product was detected by 3130xl Genetic Analyzer and GeneMapper Software (Applied Biosystems, CA, USA). For *OPRM1*, a total of 411 base pair fragments, including the first exon and a part of the first intron, were amplified. We used the same cycling conditions as with our *AVPR1a* genotyping except that we set the annealing temperature to 55 °C. We then sequenced the PCR products, both forwards and backwards, using 3130xl Genetic Analyzer (Applied Biosystems, CA, USA). In the end, we identified two novel SNPs: A111T in the first exon in nonsynonymous substitution, from leucine to phenylalanine, and T329C in the first intron. For *DAT*, we conducted PCR amplification^45^ and the length of the PCR product was detected using the same method that we used for *AVPR1a*. Primer sequences are noted in the Supplementary Methods.

### Analyses

#### Reliabilities of questionnaire items

All our analyses were conducted using version 3.4.3 of R. Using all 199 ratings, we determined the interrater reliabilities of the 54 items from the Hominoid Personality Questionnaire and the 4 subjective well-being items by computing two intraclass correlations (ICCs)^71^. One, ICC(3,1), indicates the reliability of individual ratings. The other, ICC(3,*k*), indicates the reliability of the mean of *k* ratings. We excluded items with ICC(3,1) estimates equal to or less than zero from further analyses. As in previous studies, this criterion was applied because a) the reliabilities of single items, including on human personality scales^56^, and or single behaviours in animals^72^, tend to be low, and b) items that do not capture anything about individual differences related to personality will not describe personality factors^65^.

#### Factor analyses

Using the mean of the ratings for the 77 marmosets (the aggregated data), we used the fa.parallel and fa functions from the psych package^73^ to conduct two maximum likelihood exploratory factor analyses–one for the personality items and one for the subjective well-being items. This involved our first determining the number of factors to extract by inspecting the scree plot of the eigenvalues and by testing how many factors had eigenvalues that exceed the 95^th^ percentile of eigenvalues obtained in 1000 random data sets^74^. We then rotated the factors using an orthogonal (varimax) and oblique (promax) procedure. If the oblique rotation yielded factors that were substantially correlated and a factor structure that differed markedly from orthogonally rotated factors, then we interpreted the oblique solution. If the two rotations produced similar factors, we interpreted the orthogonal solution.

We defined salient loadings as those that were equal to or exceeded |0.4|. We used structures obtained in previous studies, and especially those for common marmosets^3,4^, as a guide to labelling the factors. Also, for further analyses we created factor scores by weighting salient positive item loadings + 1, salient negative item loadings −1, and non-salient item loadings 0^51^. If an item had a salient loading on two or more factors, we assigned the weight to the factor on which it had the highest absolute loading. Unit-weighted factor scores such as these are desirable because they generalize more across samples and are highly correlated with factor scores based on exact loadings^51^.

#### Reliabilities of personality and subjective well-being factors

After creating factor scores, we computed the interrater reliabilities and internal consistencies of factors. Computer interrater reliabilities involved using all 199 ratings and computing the internal consistencies involved using the 77 aggregated ratings. Interrater reliabilities were determined by computing ICC(3,1) and ICC(3,*k*) for each factor. The internal consistency reliabilities were determined by computing Cronbach’s alpha by means of the alpha function from the psych package^73^.

#### Personality, subjective well-being, and cortisol associations

We used Pearson correlation coefficients to examine associations between personality domains and both the subjective well-being factor and the four subjective well-being items. The correlations of personality with subjective well-being measures were based on aggregated data from 77 marmosets. We corrected each set of correlations for multiple tests by using the Bonferroni procedure. The critical alpha for correlations between personality and the subjective well-being factor was equal to 0.05/3. The critical alpha for correlations between personality and the subjective well-being items was equal to 0.05/12. The correlations of personality and subjective well-being with hair cortisol level were based on aggregated data from 53 marmosets with complete personality, subjective well-being, and cortisol data. We adjusted for multiple tests and the critical alpha for these correlations was therefore equal to 0.05/4.

To test whether the sex skew and/or non-normal rearing or the large number of subjects that were not reared by their parents or not housed with conspecifics distorted these associations, we conducted robustness checks. This involved our fitting linear regressions. These analyses were limited to 68 males when examining associations between personality and the subjective well-being measures and to 52 males when examining associations between cortisol and either personality or subjective well-being. The response variable was either one of the subjective well-being variables or hair cortisol level. In addition to the main effects of one of the personality domain scores, these analyses included a main effect indicating whether an individual was parent-reared and housed with conspecifics ( + 1) or whether they were not (−1), and an interaction that tested whether the size or direction of the association varied as a function of a normal rearing and housing history.

The critical test in each analysis was whether the interaction effect was significant. After adjusting for multiple tests, the critical alpha for regressions in which the subjective well-being factor was the response variable was 0.05/3, the critical alpha for regressions in which the response variable was one of the subjective well-being items was 0.05/12, and the critical alpha for regressions in which the response variable was cortisol level was 0.05/4.

#### Genetic associations

To test for associations between genotype and the personality factors or cortisol, we used the MCMCglmm package in R^75^ to fit animal models for each response variable. An animal model is a multilevel model that includes relatedness as a random effect^76^. Animal models thus provide heritability estimates and eliminate the possibility that fixed genotype effects are confounded by the fact that related individuals are more likely to resemble one another and inherit the same genetic polymorphisms.

We excluded females from these analyses and assumed an additive mode of inheritance. For *AVPR1a*, LL individuals served as the reference group and were compared to SL individuals and to SS individuals. For *OPRM1* and *DAT*, we compared individuals that were homozygous for the more common genotype to a combined sample of individuals that were homozygous for the rare genotype and individuals that were heterozygous.

We fit three models for each response variable (a personality factor or hair cortisol level). The first model included the random relatedness effects and fixed genotype effects. The second model included the same effects as the first model and a variable that indicated whether an individual was parent-reared and housed with conspecifics (1 = yes, −1 = no) as a fixed effect. The third model included the same effects as the second model and interaction terms as fixed effects. The interaction terms were used to test whether the genotype effect varied as a function of subjects’ backgrounds. We used the deviance information criteria to identify the model that had the best balance of fit and parsimony and interpreted this model. Further details on model fitting are available in the Supplementary Methods.

### Data availability statement

The datasets generated during and/or analysed during the current study are available from the corresponding author on reasonable request.

## Electronic supplementary material

Supplementary methods, tables, and results
