# Supplementary material for: Common marmoset (Callithrix jacchus) personality, subjective well-being, hair cortisol level and AVPR1a, OPRM1, and DAT genotypes
Source: Sci Rep. 2018 Jul 6;8:10255. doi: 10.1038/s41598-018-28112-7 (PMC6035208; doi:10.1038/s41598-018-28112-7)
Supplement: Supplementary file 1 — Supplementary methods, tables, and results [file 41598_2018_28112_MOESM1_ESM.docx]

Common marmoset (*Callithrix jacchus*) personality, subjective well-being, hair cortisol level and *AVPR1a*, *OPRM1*, and *DAT* genotypes

Miho Inoue-Murayama^1,2^, Chihiro Yokoyama^3^, Yumi Yamanashi^4,1^, Alexander Weiss^5,6,*^

^1^Wildlife Research Center of Kyoto University, Kyoto, Japan

^2^Wildlife Genome Collaborative Research Group, National Institute for Environmental Studies, Tsukuba, Japan

^3^Functional Architecture Imaging Team, RIKEN Center for Life Science Technologies, Kobe, Japan

^4^Center for Research and Education of Wildlife, Kyoto City Zoo, Kyoto, Japan

^5^School of Philosophy, Psychology and Language Sciences, Department of Psychology, The University of Edinburgh, United Kingdom

^6^Scottish Primate Research Group

^*^Corresponding author at: Department of Psychology; School of Philosophy, Psychology and Language Sciences; The University of Edinburgh; 7 George Square; Edinburgh EH8 9JZ; United Kingdom; Tel: +44 131 650 3456. Email: [alex.weiss@ed.ac.uk](mailto:alex.weiss@ed.ac.uk)

Note: All authors contributed equally to this work.

**Supplementary Methods**

**Subjects**

Animal husbandry practices at the RIKEN Center for Lifestyle Technologies were as follows. On the day of their birth, animals were left in their parents’ care to breastfeed. Animals that were not reared by their parents, for example in the event of the birth of triplets or parental neglect, were separated from their parents the day after they are born and hand-reared in climate-controlled rearing cages by human caregivers. Hand-reared animals were bottle-fed 5 times per day from 1 to 7 days after birth, 4 times per day up to 21 days after birth, and then 3 times per day up to 28 days after birth. At 28 days after birth, weaning food was started in addition to twice daily bottle-feeding. The animals were then weaned from 50 to 70 post-birth. A thermal insulation box and a towel roll in the box were used for rearing from 1 to 21 days after an animal was born. From 21-days post-birth to weaning, the animals were housed in a wire-mesh box sized 390 × 230 × 300 mm furnished with a hammock, perches, a towel roll, a feeding dish, and a water bottle. After weaning, the animals began living in a home cage with age peers in the breeding room. Between 6 and 12 months of age, animals who were reared by their parents left their family cages and began living independently in one of the home cages.

Subjects were housed in breeding rooms that had a 12-h light-dark cycle (light: 08:00–20:00). Each enclosure (1630 × 760 × 831 mm for families, 660 × 650 × 600 or 660 × 450 × 600 mm for pairs or individuals) had several wooden perches, a plastic cube-shaped shelter, a food tray and a water dispenser. The temperature and humidity in the breeding room were maintained at approximately 28°C and 50%, respectively. Twice daily, subjects received solid food (CMS-1, CLEA Japan, Inc., Tokyo, Japan) mixed with an appropriate amount of powdered milk formula, honey, gluconic acid, calcium, vitamin C and lactobacillus probiotic. Once per week the subjects’ diets were supplemented with chopped boiled eggs or bananas.

**Personality and subjective well-being ratings**

The Hominoid Personality Questionnaire consists of 54 items, each comprising a personality-descriptive adjective and a one to three sentence that sets that adjective in the context of primate behaviour^1,2^. For example, the item ‘fearful’ is: “**FEARFUL**: Subject reacts excessively to real or imagined threats by displaying behaviours such as screaming, grimacing, running away or other signs of anxiety or distress.”

The four subjective well-being questionnaire items were based on aspects of subjective well-being previously in humans^3^. The first item on the subjective well-being questionnaire asked raters to report the degree to which the subject experiences positive versus negative affect.

Estimate the amount of time the monkey is happy, contented, enjoying itself, or otherwise in a positive mood. Assume that at other times the monkey is unhappy, bored, frightened, or otherwise in a negative mood.

The second item asked raters to report how much pleasure the subject derives from social interactions.

Estimate the extent to which social interactions with other monkeys are

satisfying, enjoyable experiences as opposed to being a source of fright,

distress, frustration, or some other negative experience. It is not the

number of social interactions that should be estimated, but the extent to

which social interactions that do occur are a positive experience for the

monkey. Use as many social interactions that you can recall as a basis for

your judgment.

The third item asked raters to indicate the extent to which the subject can achieve its goals.

Estimate, for this monkey, the extent to which it is effective or successful in

achieving its goals or wishes. Examples of goals would be achieving desired

locations, devices, or materials in the enclosure. Keep in mind that each

monkey will presumably have its own set of goals that may be different

from other monkeys.

The fourth item was designed to get raters to indicate each subjects’ global life satisfaction.

Imagine how happy you would be if you were that monkey for a week. You would be exactly like that monkey. You would behave the same way as that monkey, would perceive the world the same way as that monkey, and would feel things the same way as that monkey.”.

Both questionnaires instruct raters to not discuss their ratings. Furthermore, responses to items on both questionnaires were made on a seven-point scale. For the personality questionnaire, “1” was defined as “Displays either total absence or negligible amounts of the trait.”; “7” was defined as (“Displays extremely large amounts of the trait.”). For the subjective well-being scale, “1” was defined as “Displays either total absence or negligible amounts of the trait or state.”; “7” was defined as “Displays extremely large amounts of the trait.”.

For this study, we used Japanese-language versions of the questionnaires created using a back-translation procedure. A previous study showed that the psychometric properties of the Japanese-language versions of these questionnaires were comparable to those of the original English-language scales^1^. The Japanese- and English-language questionnaires are available at <http://extras.springer.com/2011/978-1-4614-0175-9/>.

**Cortisol**

To determine cortisol concentration, we first washed the samples by shaking test tubes containing 3 ml isopropanol and the samples three times for 2 minutes each. After drying samples in a vacuum oven at 38°C overnight, samples were stored at ambient temperature until analysis. We then weighed out about 15 mg of the samples and cut them into sections ranging from 3-4 mm in length. Next, we placed these samples into 2 ml tubes into which we added 3 ml of methanol. Cortisol was extracted by shaking tubes for 17 hours at ambient temperature. Following extraction, samples were centrifuged, and 1 ml of supernatant was aliquoted into different tubes and evaporated in the vacuum oven at 80°C. Samples were then reconstituted using 200μl of a phosphate buffer, and cortisol concentrations were measured using a commercially available enzyme immunoassay kit (Salivary cortisol, Salimetrics LLC, Philadelphia, PA, USA). We confirmed parallelism between cortisol standards and serially diluted hair extracts by analysis of variance. A non-significant interaction between absorbance and group (standard or serially diluted hair cortisol) indicated that there was parallelism. We did not find any interaction between cortisol standards and diluted hair cortisol (F = 0.061, df = 1, P = 0.810).

**Primers for genotyping**

To amplify *AVPR1a* we used the forward primer 5’-ATGTGGTCTGTCTGGGATGC-3’ and the reverse primer 5’-GGGTGCGACTGTAGTACACA-3’, both of which were designed based on the common marmoset genome sequence (ENSCJAG00000006111). To amplify *OPRM1* we used the forward primate 5’-GCTTGGAACCCGAAAAGTC-3’ and the reverse primer 5’-GAGTTAGGTGTCTCTTTGTACC-3’. Both primers were designed based on the genome sequence of the common marmoset (ENSCJAG00000012747). To amplify *DAT* we used the forward primer 5’-TGTGGGTGCTTGTGGTGTAG-3’ and the reverse primer 5’-AGGCCAGGCAGAGTGTGG-3’^4^.

**Animal models**

The random effect of relatedness was based on a pedigree of 142 individuals, which included 91 maternities and paternities, 227 full siblings, 228 maternal siblings, 236 paternal siblings, 69 maternal grandmothers and grandfathers, and 69 paternal grandmothers and grandfathers. Models 1, 2, and 3 for each phenotype included fixed effects for one candidate gene. For *AVPR1a*, these fixed effects included a variable that compared SL to LL subjects and a variable that compared SS to LL subjects. For *OPRM1* A111T, this fixed effect compared AA subjects to all other subjects. For *OPRM1* T329C, this fixed effect compared CC subjects to all other subjects. For *DAT*, this fixed effect compared subjects who carried the 420 bp allele to all other subjects. Model 2 also included background as a fixed effect: parent-reared subjects that were socially housed were coded +1 and all other subjects were coded -1. Model 3 also included the background × rearing interaction terms.

Because all three models included random effects of relatedness, we were able to estimate the heritability of each personality domain by computing the ratio of the variance attributable to the random effects for the animal (animal effects) over the sum of these effects and the residual variance. The MCMCglmm function that we used to fit these models uses Markov Chain Monte Carlo estimation to determine the parameters of a posterior distribution and an inverse-Gamma distribution as the prior for variance components^5^. For our models, we specified priors with a belief parameter (ν) of 0.75 and a covariance matrix (**V**) of 0.5. We ran the models for 50,000,000 iterations, had a burn-in period of 10,000,000, and thinned the samples from the posterior distribution to 4000.

References

1 Weiss, A. *et al.* Assessing chimpanzee personality and subjective well-being in Japan. *Am. J. Primatol.* **71**, 283-292, doi:10.1002/ajp.20649 (2009).

2 Weiss, A. in *Personality in Nonhuman Animals* (eds J. Vonk, A. Weiss, & S. Kuczaj) 19-38 (Springer, 2017).

3 King, J. E. & Landau, V. I. Can chimpanzee (*Pan troglodytes*) happiness be estimated by human raters? *J. Res. Pers.* **37**, 1-15, doi:10.1016/S0092-6566(02)00527-5 (2003).

4 Lucarelli, M. *et al.* Polymorphism of the 3'-UTR of the dopamine transporter gene (*DAT*) in New World monkeys. *Primates*, doi:10.1007/s10329-016-0560-0 (2016).

5 Hadfield, J. MCMC methods for Multi-response Generalized Linear Mixed Models: The MCMCglmm R Package. *J. Stat. Softw.* **33**, 1-22 (2010).

**Supplementary Tables**

Table S1: Interrater reliabilities of personality and subjective well-being items

| Items | ICC(3,1) | ICC(3,*k*) |
| --- | --- | --- |
| **Personality** |  |  |
| Solitary | 0.46 | 0.69 |
| Protective | 0.46 | 0.69 |
| Sympathetic | 0.42 | 0.65 |
| Sociable | 0.40 | 0.63 |
| Gentle | 0.37 | 0.60 |
| Imitative | 0.37 | 0.60 |
| Helpful | 0.35 | 0.58 |
| Individualistic | 0.33 | 0.56 |
| Affectionate | 0.33 | 0.56 |
| Irritable | 0.30 | 0.52 |
| Friendly | 0.27 | 0.49 |
| Aggressive | 0.25 | 0.46 |
| Impulsive | 0.25 | 0.47 |
| Excitable | 0.24 | 0.45 |
| Submissive | 0.24 | 0.45 |
| Dominant | 0.23 | 0.44 |
| Curious | 0.22 | 0.42 |
| Independent | 0.22 | 0.42 |
| Stable | 0.20 | 0.39 |
| Autistic | 0.20 | 0.39 |
| Stingy/greedy | 0.20 | 0.39 |
| Active | 0.20 | 0.40 |
| Erratic | 0.20 | 0.39 |
| Disorganized | 0.20 | 0.39 |
| Fearful | 0.19 | 0.38 |
| Inquisitive | 0.19 | 0.38 |
| Bullying | 0.18 | 0.36 |
| Distractible | 0.16 | 0.32 |
| Predictable | 0.16 | 0.33 |
| Thoughtless | 0.15 | 0.31 |
| Jealous | 0.14 | 0.30 |
| Playful | 0.14 | 0.29 |
| Conventional | 0.14 | 0.30 |
| Clumsy | 0.14 | 0.29 |
| Dependent/follower | 0.13 | 0.27 |
| Vulnerable | 0.11 | 0.24 |
| Cool | 0.11 | 0.25 |
| Intelligent | 0.11 | 0.23 |
| Timid | 0.10 | 0.23 |
| Decisive | 0.10 | 0.23 |
| Cautious | 0.09 | 0.20 |
| Sensitive | 0.09 | 0.19 |
| Defiant | 0.07 | 0.17 |
| Lazy | 0.07 | 0.16 |
| Manipulative | 0.04 | 0.09 |
| Innovative | 0.03 | 0.08 |
| Depressed | 0.03 | 0.08 |
| Reckless | -0.01 | -0.04 |
| Inventive | -0.01 | -0.02 |
| Unemotional | -0.05 | -0.14 |
| Persistent | -0.06 | -0.19 |
| Quitting | -0.06 | -0.16 |
| Anxious | -0.08 | -0.23 |
| Unperceptive | -0.09 | -0.26 |
|  |  |  |
| **Subjective well-being** |  |  |
| Balance of moods | 0.14 | 0.30 |
| Ability to achieve goals | 0.14 | 0.30 |
| Be marmoset | 0.10 | 0.23 |
| Pleasure from social interactions | 0.07 | 0.16 |

Note. ICC(3,1) and ICC(3,*k*) indicate the reliability of individual ratings and mean ratings, respectively.

Table S2: Promax-rotated factor loadings and the interfactor correlations

|  | Dominance | Sociability | Neuroticism | h^2^ |
| --- | --- | --- | --- | --- |
| Defiant | **0.89** | 0.01 | -0.21 | 0.748 |
| Stingy/greedy | **0.87** | -0.07 | -0.14 | 0.744 |
| Jealous | **0.86** | -0.04 | -0.15 | 0.725 |
| Aggressive | **0.84** | -0.21 | -0.26 | 0.752 |
| Dominant | **0.82** | -0.17 | -0.19 | 0.701 |
| Bullying | **0.80** | -0.13 | -0.19 | 0.652 |
| Irritable | **0.80** | 0.07 | 0.12 | 0.668 |
| Excitable | **0.75** | 0.19 | 0.34 | 0.719 |
| Submissive | **-0.74** | 0.11 | **0.41** | 0.591 |
| Impulsive | **0.71** | 0.15 | **0.43** | 0.756 |
| Disorganized | **0.67** | 0.13 | 0.35 | 0.616 |
| Cool | **-0.66** | -0.02 | -0.31 | 0.620 |
| Gentle | **-0.65** | **0.48** | 0.01 | 0.786 |
| Friendly | **-0.65** | **0.50** | 0.00 | 0.820 |
| Erratic | **0.65** | -0.02 | 0.32 | 0.626 |
| Active | **0.63** | **0.58** | -0.01 | 0.577 |
| Manipulative | **0.62** | 0.02 | **-0.55** | 0.539 |
| Predictable | **-0.47** | -0.03 | -0.18 | 0.276 |
| Conventional | **-0.45** | **0.40** | -0.06 | 0.477 |
| Thoughtless | 0.39 | 0.09 | 0.24 | 0.226 |
| Distractible | 0.39 | 0.03 | 0.32 | 0.297 |
| Dependent/follower | -0.26 | **0.76** | 0.26 | 0.617 |
| Imitative | -0.03 | **0.74** | 0.02 | 0.544 |
| Helpful | -0.28 | **0.72** | -0.11 | 0.774 |
| Independent | 0.36 | **-0.71** | -0.12 | 0.668 |
| Solitary | 0.17 | **-0.70** | 0.21 | 0.742 |
| Protective | -0.27 | **0.69** | -0.04 | 0.664 |
| Individualistic | 0.32 | **-0.67** | 0.10 | 0.723 |
| Sympathetic | **-0.41** | **0.63** | 0.02 | 0.661 |
| Playful | 0.27 | **0.62** | 0.08 | 0.361 |
| Affectionate | **-0.49** | **0.61** | 0.14 | 0.662 |
| Sociable | **-0.45** | **0.60** | -0.21 | 0.875 |
| Curious | **0.44** | **0.59** | 0.01 | 0.428 |
| Inquisitive | 0.39 | **0.54** | 0.02 | 0.346 |
| Sensitive | **-0.41** | **0.49** | -0.16 | 0.610 |
| Lazy | **-0.42** | **-0.46** | 0.32 | 0.460 |
| Innovative | 0.18 | **0.41** | 0.02 | 0.166 |
| Timid | -0.04 | 0.00 | **0.77** | 0.580 |
| Autistic | -0.11 | -0.01 | **0.65** | 0.402 |
| Fearful | 0.13 | 0.13 | **0.59** | 0.351 |
| Stable | -0.32 | 0.22 | **-0.56** | 0.674 |
| Vulnerable | **-0.43** | -0.10 | **0.56** | 0.424 |
| Clumsy | -0.04 | -0.15 | **0.44** | 0.261 |
| Intelligent | -0.15 | 0.35 | **-0.42** | 0.487 |
| Depressed | -0.23 | -0.36 | **0.40** | 0.386 |
| Cautious | 0.03 | 0.07 | 0.30 | 0.082 |
| Decisive | -0.21 | 0.26 | -0.28 | 0.300 |
| Proportion of variance | 0.28 | 0.18 | 0.10 |  |
| Interfactor Correlations | | | | |
|  | Dominance | Sociability | Neuroticism |  |
| Dominance | 1.00 | -0.22 | 0.23 |  |
| Sociability | -0.22 | 1.00 | -0.40 |  |
| Neuroticism | 0.23 | -.040 | 1.00 |  |

Note. Salient loadings, that is loadings greater than or equal to |.4| are in boldface. *h*^2^ = communalities.

Table S3: Hardy-Weinberg equilibrium tests

|  | *AVPR1a* | |  | *OPRM1* A111T | |  | *OPRM1* T329C | |  | *DAT* | |
| --- | --- | --- | --- | --- | --- | --- | --- | --- | --- | --- | --- |
| **Test** | Statistic | P |  | Statistic | P |  | Statistic | P |  | Statistic | P |
| Chi-square | 0.02 | 0.90 |  | 0.33 | 0.57 |  | 0.27 | 0.60 |  | 0.64 | 0.43 |
| Chi-square with continuity correction | 0.01 | 0.93 |  | 0.16 | 0.69 |  | 0.11 | 0.75 |  | 0.37 | 0.54 |
| Likelihood-ratio | 0.02 | 0.90 |  | 0.33 | 0.57 |  | 0.27 | 0.60 |  | 0.64 | 0.42 |
| Exact with SELOME P-value | --- | 1.00 |  | --- | 0.64 |  | --- | 0.60 |  | --- | 0.62 |
| Exact with DOST P-value | --- | 1.00 |  | --- | 0.69 |  | --- | 0.74 |  | --- | 0.63 |
| Exact with mid P-value | --- | 0.91 |  | --- | 0.57 |  | --- | 0.52 |  | --- | 0.54 |
| Permutation | 0.02 | 1.00 |  | 0.33 | 0.64 |  | 0.27 | 0.79 |  | 0.64 | 0.47 |

Note. SELOME = sum equally likely or more extreme; DOST = double one-sided tail probability.

Table S4

Model fit indices and heritabilities for personality domains and cortisol.

|  | Dominance | | | | Sociability | | | | Neuroticism | | | | Cortisol | | | |
| --- | --- | --- | --- | --- | --- | --- | --- | --- | --- | --- | --- | --- | --- | --- | --- | --- |
| Model | DIC | h2 | L95 | U95 | DIC | h2 | L95 | U95 | DIC | h2 | L95 | U95 | DIC | h2 | L95 | U95 |
| AVPR1a |  |  |  |  |  |  |  |  |  |  |  |  |  |  |  |  |
| Model 1 | 464.3431 | 0.38 | 0.04 | 0.82 | 441.4149 | 0.52 | 0.11 | 0.94 | 449.3675 | 0.38 | 0.05 | 0.83 | 363.5651 | 0.36 | 0.04 | 0.81 |
| Model 2 | 464.9358 | 0.39 | 0.05 | 0.82 | 437.8701 | 0.53 | 0.11 | 0.93 | 426.0713 | 0.54 | 0.11 | 0.92 | 365.1403 | 0.36 | 0.04 | 0.82 |
| Model 3 | 440.9017 | 0.53 | 0.13 | 0.93 | 428.9969 | 0.56 | 0.12 | 0.94 | 420.3860 | 0.58 | 0.15 | 0.94 | 368.7809 | 0.35 | 0.04 | 0.80 |
| OPRM1 A111T |  |  |  |  |  |  |  |  |  |  |  |  |  |  |  |  |
| Model 1 | 474.1610 | 0.39 | 0.05 | 0.82 | 444.8375 | 0.61 | 0.18 | 0.96 | 454.5434 | 0.53 | 0.12 | 0.93 | 366.0763 | 0.31 | 0.03 | 0.76 |
| Model 2 | 475.9045 | 0.39 | 0.04 | 0.81 | 442.8509 | 0.60 | 0.15 | 0.95 | 437.5767 | 0.60 | 0.18 | 0.95 | 367.2813 | 0.31 | 0.03 | 0.75 |
| Model 3 | 476.8785 | 0.40 | 0.04 | 0.82 | 443.1226 | 0.59 | 0.15 | 0.95 | 434.0541 | 0.61 | 0.20 | 0.95 | 365.3751 | 0.27 | 0.03 | 0.66 |
| OPRM1 T329C |  |  |  |  |  |  |  |  |  |  |  |  |  |  |  |  |
| Model 1 | 478.6632 | 0.37 | 0.05 | 0.82 | 437.6205 | 0.67 | 0.25 | 0.97 | 454.1695 | 0.57 | 0.16 | 0.95 | 364.0808 | 0.36 | 0.04 | 0.82 |
| Model 2 | 479.7912 | 0.38 | 0.05 | 0.82 | 434.9752 | 0.67 | 0.24 | 0.97 | 435.2833 | 0.64 | 0.22 | 0.95 | 365.2809 | 0.37 | 0.03 | 0.82 |
| Model 3 | 473.6952 | 0.42 | 0.05 | 0.85 | 433.6147 | 0.67 | 0.25 | 0.97 | 437.0384 | 0.63 | 0.21 | 0.96 | 366.4868 | 0.36 | 0.04 | 0.82 |
| DAT 420 |  |  |  |  |  |  |  |  |  |  |  |  |  |  |  |  |
| Model 1 | 484.4643 | 0.39 | 0.04 | 0.84 | 447.0559 | 0.65 | 0.23 | 0.96 | 454.5109 | 0.61 | 0.19 | 0.96 | 368.7138 | 0.40 | 0.04 | 0.85 |
| Model 2 | 484.0509 | 0.39 | 0.04 | 0.84 | 447.9168 | 0.63 | 0.20 | 0.97 | 445.6092 | 0.64 | 0.21 | 0.96 | 367.7307 | 0.42 | 0.05 | 0.87 |
| Model 3 | 485.3393 | 0.40 | 0.04 | 0.85 | 446.2773 | 0.65 | 0.22 | 0.97 | 449.1009 | 0.62 | 0.18 | 0.96 | 370.2290 | 0.40 | 0.05 | 0.84 |

Note. Models in boldface had the lowest deviance information criterion (DIC) and thus represented the models with the best fit and parsimony. h2 = heritability, L95 = lower 95% credible interval, U95 = upper 95% credible interval

**Supplementary Results**

The R output for regressions to test for the robustness of the correlations between personality and subjective well-being variables are below. F1 refers to Dominance, F2 refers to Sociability, and F3 refers to Neuroticism. Parameter estimates are standardized (mean = 0, SD = 1).

**Response variable: Subjective well-being**

Coefficients:

Estimate Std. Error t value Pr(>|t|)

(Intercept) -0.04784 0.12955 -0.369 0.713

scale(F1) -0.03403 0.12482 -0.273 0.786

normal 0.10889 0.12955 0.841 0.404

scale(F1):normal -0.14545 0.12482 -1.165 0.248

Residual standard error: 1.006 on 64 degrees of freedom

Multiple R-squared: 0.03414, Adjusted R-squared: -0.01113

F-statistic: 0.7541 on 3 and 64 DF, p-value: 0.524

Coefficients:

Estimate Std. Error t value Pr(>|t|)

(Intercept) -0.003248 0.109755 -0.030 0.976

scale(F2) 0.587915 0.110614 5.315 1.44e-06 ***

normal -0.043424 0.109755 -0.396 0.694

scale(F2):normal 0.072204 0.110614 0.653 0.516

---

Signif. codes: 0 ‘***’ 0.001 ‘**’ 0.01 ‘*’ 0.05 ‘.’ 0.1 ‘ ’ 1

Residual standard error: 0.8135 on 64 degrees of freedom

Multiple R-squared: 0.3679, Adjusted R-squared: 0.3383

F-statistic: 12.42 on 3 and 64 DF, p-value: 1.694e-06

Coefficients:

Estimate Std. Error t value Pr(>|t|)

(Intercept) -0.01358 0.12234 -0.111 0.912

scale(F3) -0.50549 0.11584 -4.364 4.76e-05 ***

normal -0.06483 0.12234 -0.530 0.598

scale(F3):normal -0.10549 0.11584 -0.911 0.366

---

Signif. codes: 0 ‘***’ 0.001 ‘**’ 0.01 ‘*’ 0.05 ‘.’ 0.1 ‘ ’ 1

Residual standard error: 0.8886 on 64 degrees of freedom

Multiple R-squared: 0.2457, Adjusted R-squared: 0.2103

F-statistic: 6.949 on 3 and 64 DF, p-value: 0.0004032

**Response variable: Balance of positive and negative moods**

Coefficients:

Estimate Std. Error t value Pr(>|t|)

(Intercept) 0.01342 0.12989 0.103 0.918

scale(F1) -0.06739 0.12515 -0.538 0.592

normal -0.07645 0.12989 -0.589 0.558

scale(F1):normal -0.13050 0.12515 -1.043 0.301

Residual standard error: 1.008 on 64 degrees of freedom

Multiple R-squared: 0.02902, Adjusted R-squared: -0.0165

F-statistic: 0.6375 on 3 and 64 DF, p-value: 0.5936

Coefficients:

Estimate Std. Error t value Pr(>|t|)

(Intercept) 0.02469 0.12057 0.205 0.838402

scale(F2) 0.43136 0.12151 3.550 0.000728 ***

normal -0.17759 0.12057 -1.473 0.145661

scale(F2):normal 0.13678 0.12151 1.126 0.264503

---

Signif. codes: 0 ‘***’ 0.001 ‘**’ 0.01 ‘*’ 0.05 ‘.’ 0.1 ‘ ’ 1

Residual standard error: 0.8936 on 64 degrees of freedom

Multiple R-squared: 0.2372, Adjusted R-squared: 0.2015

F-statistic: 6.634 on 3 and 64 DF, p-value: 0.0005679

Coefficients:

Estimate Std. Error t value Pr(>|t|)

(Intercept) -0.02128 0.12702 -0.167 0.86751

scale(F3) -0.37994 0.12028 -3.159 0.00242 **

normal -0.18385 0.12702 -1.447 0.15267

scale(F3):normal -0.24652 0.12028 -2.050 0.04451 *

---

Signif. codes: 0 ‘***’ 0.001 ‘**’ 0.01 ‘*’ 0.05 ‘.’ 0.1 ‘ ’ 1

Residual standard error: 0.9226 on 64 degrees of freedom

Multiple R-squared: 0.1869, Adjusted R-squared: 0.1487

F-statistic: 4.902 on 3 and 64 DF, p-value: 0.003959

**Response variable: Pleasure from social interactions**

Coefficients:

Estimate Std. Error t value Pr(>|t|)

(Intercept) -0.10216 0.12511 -0.817 0.4172

scale(F1) -0.04942 0.12054 -0.410 0.6832

normal 0.32095 0.12511 2.565 0.0127 *

scale(F1):normal 0.01936 0.12054 0.161 0.8729

---

Signif. codes: 0 ‘***’ 0.001 ‘**’ 0.01 ‘*’ 0.05 ‘.’ 0.1 ‘ ’ 1

Residual standard error: 0.9711 on 64 degrees of freedom

Multiple R-squared: 0.09928, Adjusted R-squared: 0.05705

F-statistic: 2.351 on 3 and 64 DF, p-value: 0.08055

Coefficients:

Estimate Std. Error t value Pr(>|t|)

(Intercept) -0.04739 0.10926 -0.434 0.666

scale(F2) 0.55679 0.11012 5.056 3.83e-06 ***

normal 0.17413 0.10926 1.594 0.116

scale(F2):normal -0.03732 0.11012 -0.339 0.736

---

Signif. codes: 0 ‘***’ 0.001 ‘**’ 0.01 ‘*’ 0.05 ‘.’ 0.1 ‘ ’ 1

Residual standard error: 0.8098 on 64 degrees of freedom

Multiple R-squared: 0.3736, Adjusted R-squared: 0.3442

F-statistic: 12.72 on 3 and 64 DF, p-value: 1.28e-06

Coefficients:

Estimate Std. Error t value Pr(>|t|)

(Intercept) -0.02338 0.12812 -0.182 0.8558

scale(F3) -0.25980 0.12132 -2.142 0.0360 *

normal 0.21463 0.12812 1.675 0.0988 .

scale(F3):normal 0.14060 0.12132 1.159 0.2508

---

Signif. codes: 0 ‘***’ 0.001 ‘**’ 0.01 ‘*’ 0.05 ‘.’ 0.1 ‘ ’ 1

Residual standard error: 0.9306 on 64 degrees of freedom

Multiple R-squared: 0.1728, Adjusted R-squared: 0.134

F-statistic: 4.455 on 3 and 64 DF, p-value: 0.006632

**Response variable: Ability to achieve goals**

Coefficients:

Estimate Std. Error t value Pr(>|t|)

(Intercept) -0.04816 0.12891 -0.374 0.710

scale(F1) 0.04081 0.12421 0.329 0.744

normal 0.09672 0.12891 0.750 0.456

scale(F1):normal -0.19456 0.12421 -1.566 0.122

Residual standard error: 1.001 on 64 degrees of freedom

Multiple R-squared: 0.04362, Adjusted R-squared: -0.001214

F-statistic: 0.9729 on 3 and 64 DF, p-value: 0.4111

Coefficients:

Estimate Std. Error t value Pr(>|t|)

(Intercept) -0.009746 0.123628 -0.079 0.9374

scale(F2) 0.422472 0.124596 3.391 0.0012 **

normal -0.020446 0.123628 -0.165 0.8692

scale(F2):normal 0.068297 0.124596 0.548 0.5855

---

Signif. codes: 0 ‘***’ 0.001 ‘**’ 0.01 ‘*’ 0.05 ‘.’ 0.1 ‘ ’ 1

Residual standard error: 0.9163 on 64 degrees of freedom

Multiple R-squared: 0.198, Adjusted R-squared: 0.1604

F-statistic: 5.267 on 3 and 64 DF, p-value: 0.002609

Coefficients:

Estimate Std. Error t value Pr(>|t|)

(Intercept) -0.01884 0.12450 -0.151 0.88022

scale(F3) -0.47302 0.11789 -4.012 0.00016 ***

normal -0.07125 0.12450 -0.572 0.56912

scale(F3):normal -0.12787 0.11789 -1.085 0.28214

---

Signif. codes: 0 ‘***’ 0.001 ‘**’ 0.01 ‘*’ 0.05 ‘.’ 0.1 ‘ ’ 1

Residual standard error: 0.9043 on 64 degrees of freedom

Multiple R-squared: 0.2188, Adjusted R-squared: 0.1822

F-statistic: 5.976 on 3 and 64 DF, p-value: 0.001175

**Response variable: Global satisfaction (Be marmoset)**

Coefficients:

Estimate Std. Error t value Pr(>|t|)

(Intercept) -0.013065 0.129502 -0.101 0.920

scale(F1) -0.036340 0.124775 -0.291 0.772

normal -0.007396 0.129502 -0.057 0.955

scale(F1):normal -0.178353 0.124775 -1.429 0.158

Residual standard error: 1.005 on 64 degrees of freedom

Multiple R-squared: 0.03488, Adjusted R-squared: -0.01036

F-statistic: 0.771 on 3 and 64 DF, p-value: 0.5145

Coefficients:

Estimate Std. Error t value Pr(>|t|)

(Intercept) 0.02603 0.11785 0.221 0.826

scale(F2) 0.50497 0.11878 4.251 7.05e-05 ***

normal -0.13786 0.11785 -1.170 0.246

scale(F2):normal 0.07755 0.11878 0.653 0.516

---

Signif. codes: 0 ‘***’ 0.001 ‘**’ 0.01 ‘*’ 0.05 ‘.’ 0.1 ‘ ’ 1

Residual standard error: 0.8735 on 64 degrees of freedom

Multiple R-squared: 0.2712, Adjusted R-squared: 0.237

F-statistic: 7.938 on 3 and 64 DF, p-value: 0.0001404

Coefficients:

Estimate Std. Error t value Pr(>|t|)

(Intercept) 0.0197 0.1195 0.165 0.870

scale(F3) -0.5478 0.1132 -4.841 8.53e-06 ***

normal -0.1943 0.1195 -1.626 0.109

scale(F3):normal -0.1318 0.1132 -1.164 0.249

---

Signif. codes: 0 ‘***’ 0.001 ‘**’ 0.01 ‘*’ 0.05 ‘.’ 0.1 ‘ ’ 1

Residual standard error: 0.8681 on 64 degrees of freedom

Multiple R-squared: 0.2802, Adjusted R-squared: 0.2465

F-statistic: 8.304 on 3 and 64 DF, p-value: 9.566e-05

The R output for regressions to test for the robustness of the correlations between hair cortisol level and the personality and subjective well-being variables are below. F1 refers to Dominance, F2 refers to Sociability, F3 refers to Neuroticism, and SWB refers to subjective well-being. The response variable in all cases was hair cortisol level. Parameter estimates are standardized (mean = 0, SD = 1).

Coefficients:

Estimate Std. Error t value Pr(>|t|)

(Intercept) 0.05394 0.15132 0.356 0.7231

scale(F1) -0.29013 0.15929 -1.821 0.0748 .

normal -0.04837 0.15132 -0.320 0.7506

scale(F1):normal -0.05703 0.15929 -0.358 0.7219

---

Signif. codes: 0 ‘***’ 0.001 ‘**’ 0.01 ‘*’ 0.05 ‘.’ 0.1 ‘ ’ 1

Residual standard error: 0.9872 on 48 degrees of freedom

(16 observations deleted due to missingness)

Multiple R-squared: 0.08279, Adjusted R-squared: 0.02546

F-statistic: 1.444 on 3 and 48 DF, p-value: 0.2416

Coefficients:

Estimate Std. Error t value Pr(>|t|)

(Intercept) 0.2231 0.1493 1.495 0.141508

scale(F2) 0.6264 0.1587 3.948 0.000257 ***

normal -0.2643 0.1493 -1.771 0.082969 .

scale(F2):normal -0.2798 0.1587 -1.763 0.084183 .

---

Signif. codes: 0 ‘***’ 0.001 ‘**’ 0.01 ‘*’ 0.05 ‘.’ 0.1 ‘ ’ 1

Residual standard error: 0.895 on 48 degrees of freedom

(16 observations deleted due to missingness)

Multiple R-squared: 0.2461, Adjusted R-squared: 0.199

F-statistic: 5.222 on 3 and 48 DF, p-value: 0.003351

Coefficients:

Estimate Std. Error t value Pr(>|t|)

(Intercept) 0.075931 0.164494 0.462 0.6464

scale(F3) -0.316268 0.153279 -2.063 0.0445 *

normal -0.165141 0.164494 -1.004 0.3204

scale(F3):normal 0.008737 0.153279 0.057 0.9548

---

Signif. codes: 0 ‘***’ 0.001 ‘**’ 0.01 ‘*’ 0.05 ‘.’ 0.1 ‘ ’ 1

Residual standard error: 0.9869 on 48 degrees of freedom

(16 observations deleted due to missingness)

Multiple R-squared: 0.08323, Adjusted R-squared: 0.02593

F-statistic: 1.453 on 3 and 48 DF, p-value: 0.2393

Coefficients:

Estimate Std. Error t value Pr(>|t|)

(Intercept) 0.06365 0.14333 0.444 0.65897

scale(SWB) 0.49558 0.16594 2.987 0.00443 **

normal -0.08630 0.14333 -0.602 0.54996

scale(SWB):normal -0.30115 0.16594 -1.815 0.07581 .

---

Signif. codes: 0 ‘***’ 0.001 ‘**’ 0.01 ‘*’ 0.05 ‘.’ 0.1 ‘ ’ 1

Residual standard error: 0.946 on 48 degrees of freedom

(16 observations deleted due to missingness)

Multiple R-squared: 0.1577, Adjusted R-squared: 0.1051

F-statistic: 2.996 on 3 and 48 DF, p-value: 0.03977
